# Rainbow Archimedean spiral emission from optical fibres

**DOI:** 10.1038/s41598-021-92313-w

**Published:** 2021-06-22

**Authors:** F. Mangini, M. Ferraro, M. Zitelli, V. Kalashnikov, A. Niang, T. Mansuryan, F. Frezza, A. Tonello, V. Couderc, A. B. Aceves, S. Wabnitz

**Affiliations:** 1 Department of Information Engineering (DII), University of Brescia, Via Branze 38, 25123 Brescia, Italy; 2 Department of Information Engineering, Electronics and Telecommunications (DIET), Sapienza University of Rome, Via Eudossiana 18, 00184 Rome, Italy; 3 Université de Limoges, XLIM, UMR CNRS 7252, 123 Avenue A. Thomas, 87060 Limoges, France; 4 Department of Mathematics, Southern Methodist University, 3100 Dyer St, Dallas, TX 75205 USA

**Keywords:** Optics and photonics, Applied optics, Optical physics

## Abstract

We demonstrate a new practical approach for generating multicolour spiral-shaped beams. It makes use of a standard silica optical fibre, combined with a tilted input laser beam. The resulting breaking of the fibre axial symmetry leads to the propagation of a helical beam. The associated output far-field has a spiral shape, independently of the input laser power value. Whereas, with a high-power near-infrared femtosecond laser, a visible supercontinuum spiral emission is generated. With appropriate control of the input laser coupling conditions, the colours of the spiral spatially self-organize in a rainbow distribution. Our method is independent of the laser source wavelength and polarization. Therefore, standard optical fibres may be used for generating spiral beams in many applications, ranging from communications to optical tweezers and quantum optics.

## Introduction

**Figure 1:**
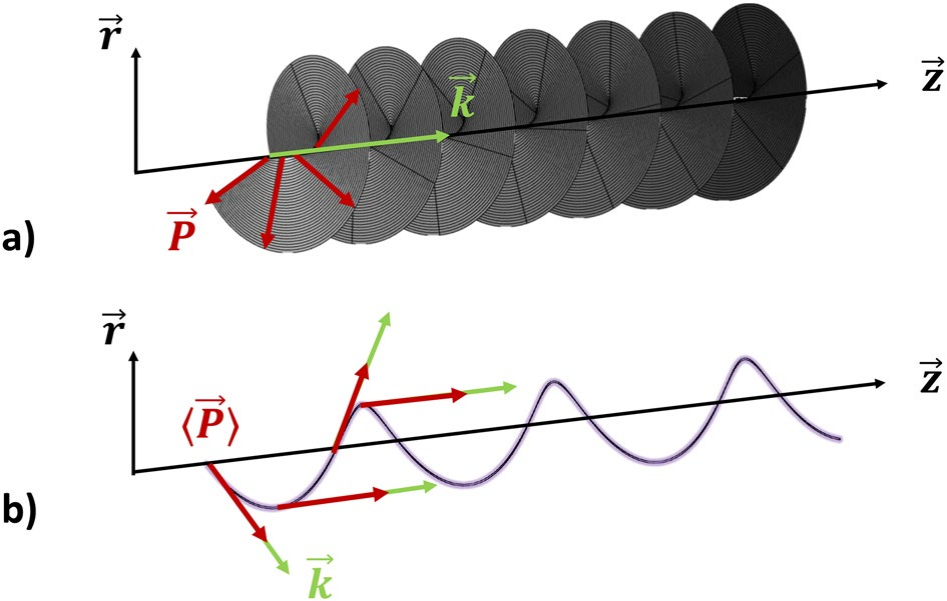
Pictorial view of (**a**) LOAM and (**b**) TOAM beam propagation.

Spiral shaped beams are conventionally obtained by means of spiral phase plates^1^ or by interfering Gaussian waves with the so-called longitudinal orbital angular momentum (LOAM) carrying beams^2^. LOAM beams are peculiar solutions of Maxwell’s equations, characterized by phase singularity and helical wavefront $\varphi$^3,4^. The main characteristic of LOAM beams is the so-called topological charge ($\ell$), an integer number that counts the number of wave front rotations over one wavelength of propagation, so that the electromagnetic field has a phase $\phi =\ell \varphi$. Early studies of the LOAM of light by Allen et al. date back to more than 30 years ago^5^. Over time, interest in LOAM beams has increased tremendously, thanks to their potential widespread applications. These range from telecommunications^6,7^ to quantum optics^8,9^, holography^10,11^, fabrication of chiral nanostructures^12,13^ and optical tweezers^14,15^, a research recognized by the award of the 2018 Nobel prize to Arthur Ashkin. LOAM beams can also be generated and propagated in fibre-based optical systems^4,16,17^. More recently, another approach of generating spiral beams via LOAM was proposed^18^. It consists of giving light a power-exponent-phase $\phi =2\pi \ell (\varphi /2\pi )^n$, being *n* a real number. In all of these cases, the orbital angular momentum is parallel to the beam wave vector, and it is therefore dubbed longitudinal^19^: $\vec {L}_l = \hbar \ell \langle \vec {k}\rangle /k$. For a LOAM beam, the Poynting vector $\vec {P}$ periodically rotates around the wave vector, as depicted in Fig. 1a.

An interesting, and somewhat less studied, case is that of beams possessing a transverse, as opposed to longitudinal, orbital angular momentum (TOAM)^19^. This type of beam can be more easily described when a ray optics approximation can be introduced, whereby we associate to the light beam a particle with a corresponding momentum $\langle \vec {P}\rangle$, which represents the average Poynting vector^19^. The resulting TOAM reads $\vec {L}_t=\vec {r}\times \langle \vec {P}\rangle$, where $\vec {r}$ is the position of the beam with respect to the origin of the reference frame. In this case, the average Poynting vector traces a helical path (see Fig. 1b).

Recall that the total angular momentum of light ($\vec {J}$) can be expressed as the sum of two contributions:

1 $$\begin{aligned} \vec {J}=\vec {S}+\vec {L} = \vec {S}+\vec {L}_l+\vec {L}_t. \end{aligned}$$

here $\vec {S}$ is the spin angular momentum (SAM) that, differently from the orbital momentum, characterizes the state of polarization of light. The SAM has attracted significant research interest, owing to its applications to nanophotonic Berry-phase devices, e.g., based on the spin Hall effect of light^20^.

Whereas LOAM beams have been mostly obtained so far by means of external optical components^2^, which poses limitations to their use in integrated optics^21^, the generation of SAM and TOAM beams can be more easily achieved^22^. The latter, in particular, can be obtained by starting from circularly polarized beams (that carry SAM), thanks to the total angular momentum conservation in the refraction or reflection of light from either dielectric^23^ or metallic interfaces^24^, where the spin-Hall effect of light (or Imbert–Fedorov shift^22^) occurs.

**Figure 2:**
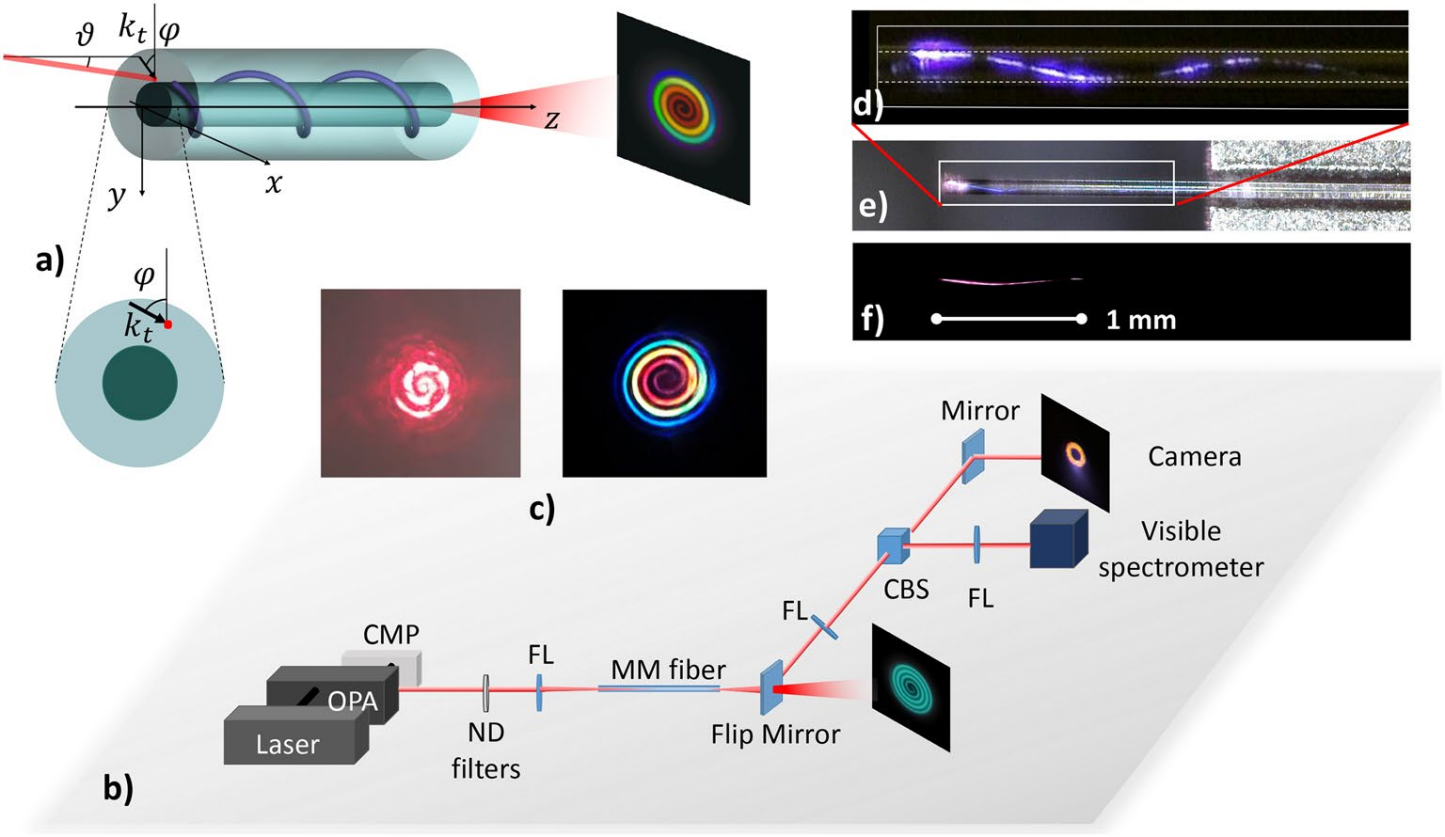
(**a**) Schematic of fibre geometry and rainbow spiral emission; $\vartheta$ is the incidence tilt of the skew ray (or zenith angle), $\varphi$ is the azimuth angle, and $k_t$ is the projection of the laser beam wave vector on the fibre transverse plane. (**b**) Schematic of set-up where typical near- and far-fields are shown. The ND filter is used to vary input power, FL stands for focus lens, and CBS for cubic beam splitter. (**c**) Spiral emission from CW He–Ne laser (left) or from femtosecond pulse laser (right). (**d**) Zoom-in of (**e**), dashed lines indicate core–cladding boundaries. (**e**) Visualization of helical beam propagation in a graded-index (GRIN) fibre, thanks to upconversion luminescence from material defects and doping. Scattered white light on the right is due to the fibre holder. (**f**) Same as (**d**) but from step-index fibre.

In this work, we propose and demonstrate how TOAM-carrying beams can be spontaneously generated in standard silica optical fibres, and seed spiral-shaped emission. As a cylindrical waveguide, an optical fibre is a natural beam shaper for TOAM beams. Our method consists of focusing a laser beam in the cladding of the fibre with proper angles $\vartheta$ and $\varphi$, as sketched in Fig. 2a. This produces an azimuthal component to the beam wave vector, so that when propagating in the fibre, the beam starts twisting along the core–cladding (or air–cladding) interface following a helical trajectory while spreading because of diffraction. Eventually, the TOAM beam is converted, at least partially, into a LOAM beam. By controlling input angles and position offset, one may optimize the trade-off between diffraction-driven spreading and helical winding. As a result, when the beam occupies the whole cladding area, it also acquires a phase which nonlinearly rotates with the azimuth angle $\varphi$, thus leading to an Archimedean spiral intensity pattern in the far-field, as shown in Fig. 2b. By our approach, we can easily change the chirality of the spiral, by just inverting the sign of the incidence angle $\vartheta$, thus flipping the winding direction. In the nonlinear propagation regime, supercontinuum (SC) generation leads to high-brightness spectral broadening, covering all visible spectrum. We reveal that in the far-field, the SC is still a spiral beam, which now contains a mixture of different colours. Under proper input coupling conditions, these colours spontaneously separate in space, giving rise to a spiral-shaped rainbow emission. We conclude by pointing out analogies with the effect of conical emission in filamentation, that accompanies high-power laser beam propagation in air^25,26^.

## Results

Our approach to generate spiral emission by means of commercial, standard telecom fibres is remarkably simple. We demonstrate that a laser beam, coupled with proper input zenith angle $\vartheta$ and azimuth angle $\varphi$ (see Fig. 2a) into the fibre, produces a spiral emission in the far-field, independently of its wavelength or polarization. The main conditions on the incidence beam angles are: small zenith angles (e.g., $\vartheta <2^\circ$), and azimuthal angles such that the projection of the incident beam wave vector on the transverse plane of the fibre ($k_t$) is tangent to the core/cladding interface, so that its radial component is vanishing (see Fig. 2a). The experimental set-up to study spiral emission is shown in Fig. 2b. As a source for SC generation, we used intense femtosecond laser pulses at the 1030 nm wavelength. As discussed in the “Supplementary”, we observed that spiral emission can be obtained with shorter wavelengths as well. As it exits the fibre, the beam is collected by an imaging lens to measure the near-field spatial profile and spectrum with a CCD camera and a spectrometer, respectively. By means of a flipping mirror we could also image the far-field.

The spiral emission shown in the left panel of Fig. 2c was obtained by means of a He–Ne CW laser. On the other hand, the true-colour picture on the right panel of the same figure was obtained by matching four different conditions: TOAM seeding, spiral emission, SC generation, and colour spatial separation. In the following, we investigate all of these elements, one at a time.

### TOAM seeding

In our experiments, we removed the external plastic coating of the fibre, so that we can directly track the beam propagation inside the fibre by naked eye, by exploiting the phenomenon of upconversion luminescence due to the presence of doping, and of intrinsic and extrinsic defects in MMFs^27,28^ (see “Methods”). We underline that TOAM-carrying beam propagation is a linear phenomenon. High-power pulses are only used in order to excite the luminescence, which is needed for tracking the path of light beams. As illustrated in Fig. 2, a helical beam path in MMFs was observed when injecting, with appropriate incidence angles, the laser beam in proximity of either the core–cladding or the cladding–air interfaces, respectively. Results in Fig. 2 refer to both step-index (SI) and graded-index (GRIN) MMFs (in the “Supplementary material” we also report the case of a singlemode fibre). Figure 2d–f shows beam paths as revealed by the multiphoton-absorption generated luminescence, which is measured by a microscope positioned on top of the MMFs.

With either GRIN or SI fibres, we may always clearly recognize a visible photoluminescence, that traces helical beam paths. These can be associated with the propagation of TOAM beams, which are internally twisting around the core–cladding or the cladding–air interfaces, respectively. It is interesting to observe the difference in colour between luminescence from either a GRIN fibre (Fig. 2d,e) or a SI fibre (Fig. 2f). This is due to the presence of doping in the core of a GRIN fibre, which produces a blue luminescence. Whereas silica intrinsic defects produce a red light at the core–cladding interface of a SI fibre^28^. For the same reason, beams tracing a helical path around the cladding–air interface shine red light from both SI and GRIN fibres. The propagation of TOAM-carrying beams with helical paths was previously reported in a glass rod^29^: it was shown that key to produce a TOAM beam is the presence of a refractive index gradient at the glass–air interface. Accordingly, we also found that the generated luminescence only traces a helical beam path when the input beam touches the inner or the outer boundaries of the fibre cladding.

**Figure 3:**
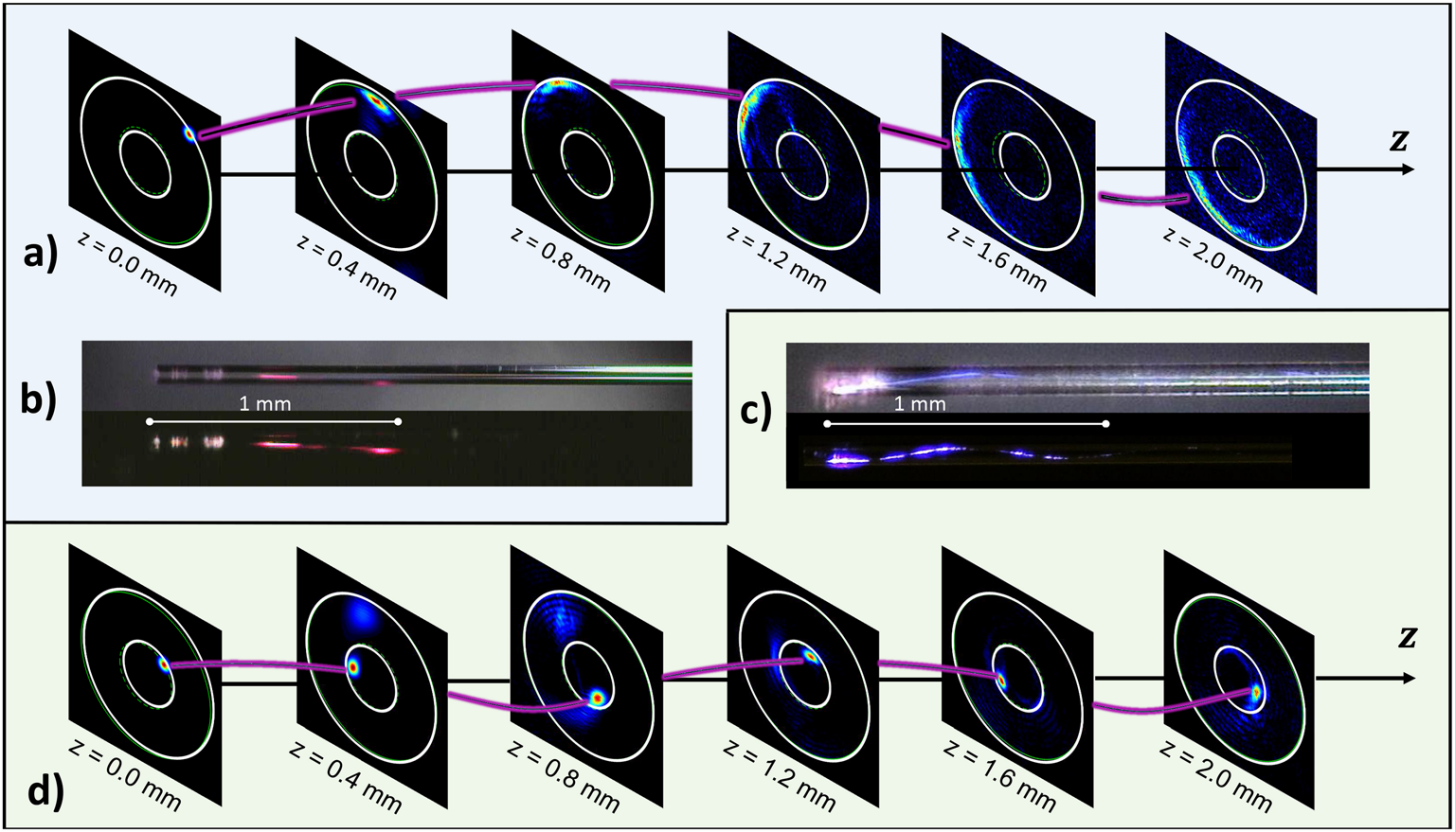
TOAM carrying beam tracking, obtained when the input beam is incident at the cladding–air (**a**,**b**) or the core–cladding interface (**c**,**d**), respectively. The purple curves in (**a**) and (**d**) represent the Poynting vector trajectory. Simulated near-field intensities are compared with experimentally observed luminescence intensities.

In order to confirm the observed TOAM carrying beam trajectories, we performed a series of numerical simulations (see “Methods” for details). In Fig. 3a,b, we illustrate the case of a beam generated at the cladding–air interface. For a comparison, the case of core–cladding interface incidence is also reported in Fig. 3c,d. In both cases, a good agreement is found between numerical and experimental values of the winding period, which grows linearly proportional to the curvature radius. To visually compare numerical and experimental results, we highlight the Poynting vector trajectory with a purple curve (similar to the one in Fig.1b) crossing the numerical simulation images at the maximum intensity points. Simulations also show that, owing to diffraction, the beam size progressively widens while internally twisting around the interface, which eventually invalidates the ray-optics approximation. Nevertheless, the total angular momentum conservation law ($\vec {J}=const.$) ensures that the TOAM beam maintains its angular momentum during the propagation^19^. In the next section, we will show how the TOAM is converted into a power-exponent-phase LOAM along its propagation, so that, under suitable conditions, the beam acquires a proper amplitude and phase, in order to produce an Archimedean spiral shaped intensity profile in the far-field.

### Spiral emission

When we observe spiral emission, the luminescence always traces a helical shape. Therefore, we can state that the spiral emission is seeded by the TOAM. In other words, generating a spiral shape in the far-field intensity requires a symmetry breaking of the propagating field (e.g., in the phase) in the plane transverse to its propagation direction, which always produces a TOAM^22,30^. Indeed, such spiral emission from multimode optical fibres was earlier observed, and explained by means of a ray tracing model^31^. In that work, a helical trajectory for the propagating beam is imposed by a controlled microbending along the fibre. Whereas, in our case, the TOAM is provided by the input coupling conditions. Remarkably, our spiral emission generation method is linear, hence it does not require particularly high laser powers. As detailed in the “Supplementary”, spiral emission could be generated independently of the laser average power and pulse duration. For example, we could generate a red spiral beam by using a CW He–Ne laser (see Fig. 2b-left), and even by using a standard laser pointer as a source (see “Supplementary”)! Furthermore, spiral emission does not appear to be affected by varying the input state of polarization, and it is wavelength-independent.

**Figure 4:**
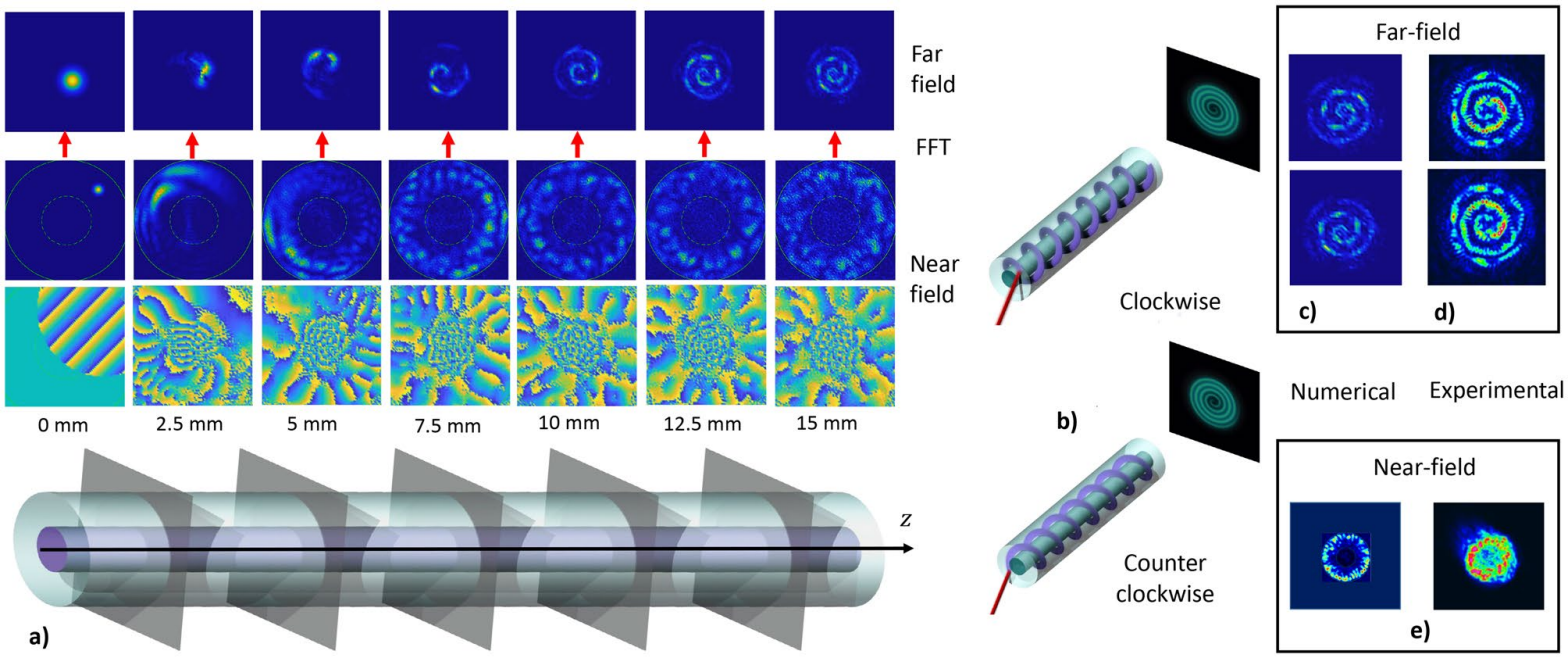
(**a**) Sketch of the first 1.5 cm of the fibre (bottom); The near-field (amplitude and phase) is numerically computed each 2.5 mm, corresponding to the position where the transverse planes cut the fibre (centre). The far-field is calculated as the Fourier transform of the near-field, showing spiral emission formation (top). (**b**) Tunability of optical chirality. The spiral emission ℓ sign depends on the input beam position with respect to the fibre centre. (**c**) Numerical confirmation of optical chirality inversion. (**d**) Experimental results corresponding to (**c**). (**e**) Comparison of numerical and experimental near-field intensities. All simulations and experiments were performed with an input peak power of 3 MW, a wavelength of 1030 nm, $\vartheta = 1.5 ^\circ$ and $\varphi = 45 ^ \circ$.

In order to theoretically reproduce the observed spiral emission, we performed extensive numerical simulations. Figure 4a shows that a beam, which is initially focused on small spot inside the fibre cladding, progressively occupies all of the cladding area, and it acquires a phase profile that rapidly varies in the azimuthal direction (see “Methods” for details of the numerical model). In Fig. 4a we also plot the far-field, which is generated by free-space propagation of the spatial field distribution at given specific points inside the fibre. Specifically, the far-field is calculated as the Fourier transform of the near-field in each slice. In the “Supplementary materials”, we provide videos of amplitude and phase evolution of both the near- and far-fields along the beam propagation. Thanks to the total orbital angular momentum conservation, the initial TOAM is converted into a LOAM. In particular, as Fig. 4a shows, the acquired phase nonlinearly scales with the angle $\varphi$. The obtained phase pattern at 5 mm of propagation is pretty similar to that of Ref.^18^.

Simulations also explain how to tune the chirality of the spiral. Depending on the beam input position, one can reverse the chirality, from clockwise to counterclockwise. This is illustrated in Fig. 4b. Diametrically opposed input positions correspond to opposite winding, hence flipping the chirality sign. Excellent qualitative agreement between simulations and experiments was achieved, as shown in Fig. 4c,d and Fig. 4e for the far- and near-field respectively. As can be seen from Fig. 4a, for a given input beam coupling configuration (angle and position offset), the only parameter determining the length of the spiral arm is the fibre length. In Fig. 2f of the “Supplementary materials” we provide the experimental confirmation of the lengthening of the spiral arms as the fibre length grows larger.

As shown in Fig. 4, the TOAM beam intensity at the fibre output facet has an annular shape, similar to the case of LOAM beams. Note that the generation of the so-called hollow beams, with a helical path around the core induced by tilted laser-fibre coupling, was previously reported^32^. However, it remained unnoticed that, besides the annular transverse intensity beam pattern, a proper phase pattern (associated with TOAM) is necessary, in order to generate spiral emission in the far-field. Therefore, the conditions for spiral emission are stricter than those required for the formation of an hollow beam. While the latter can be realized with relatively large input tilt angles and position offsets^32^, spiral emission is only obtained when the input laser beam is focused close to fibre index interfaces, and with a small tilt angle $\vartheta$ (between $1^{\circ }$ and $2^{\circ }$). We observed, both numerically and experimentally, that if the incidence is not grazing, the beam never reaches the proper amplitude and phase which are necessary to generate a spiral pattern in the output far-field intensity profile. Specifically, we noted that as $\vartheta$ increases, the range of parameters leading to spiral emission existence tends to shrink.

Another critical condition for spiral emission involves the fibre length. One can appreciate from Fig. 4a that spiral formation requires a certain minimum propagation length (estimated to be of a few millimeters with our parameters). At the same time, the fire must be short enough, in order to avoid substantial beam spreading inside the core, thus washing out the annular shape of the near-field intensity profile. We estimated, both numerically and experimentally, the optimal threshold length to be about 2 cm for $\vartheta \simeq 2^\circ$. With such short fibre lengths, we managed to keep the output transmission above 90% even at MW input powers^33^.

### SC generation

**Figure 5:**
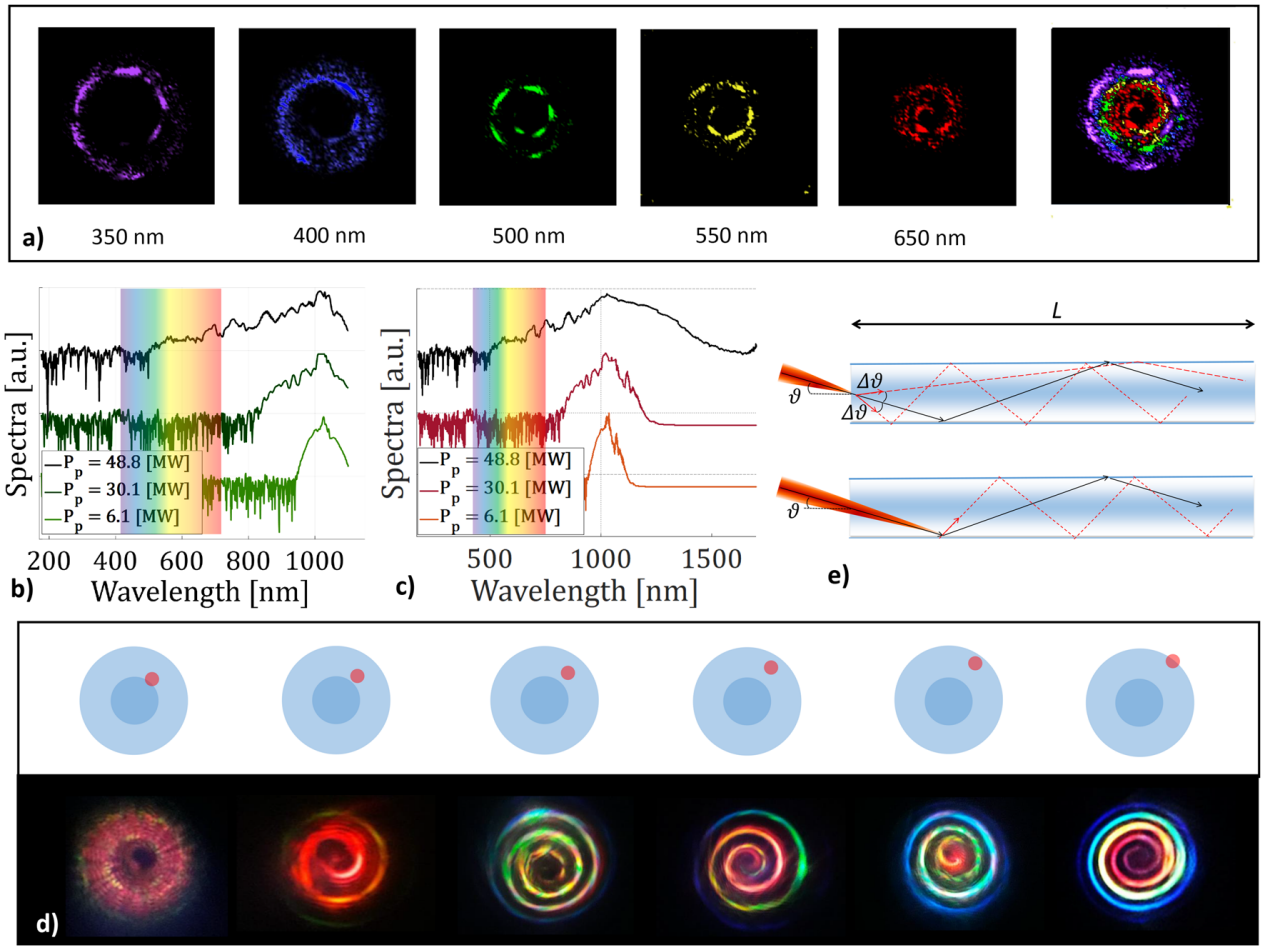
(**a**) Rainbow spiral simulation as a sum of the single color components. In all images, we only display points with intensities larger than two thirds of the peak value. (**b**, **c**) Fibre output spectra at different input peak powers (up to 48 MW) at 1030 nm in step- and graded-index fibres, respectively, keeping the same coupling conditions. (**d**) Far-field image of graded-index fibre when varying the input beam position while keeping the peak power at 48 MW, with $\vartheta = 1.5 ^\circ$ and $\varphi = 45 ^ \circ$. (**e**) Ray optics sketch of conical emission analogy. On the top, the SC is generated inside the fibre volume. On the bottom, the SC generates at the air–cladding interface.

Whenever the injected power of a near-infrared femtosecond laser source was high enough to produce SC generation, i.e., a wide nonlinear spectral broadening of the input laser pulses, a coloured spiral intensity profile in the far-field was emitted from the fibre output facet. In Fig. 5b,c we report examples of spectra obtained from SI and GRIN MMFs, respectively, when varying the input peak power. When the latter reaches 48 MW, the whole visible spectral range is covered, producing all of the images shown in Fig. 5d. Due to the absence of clear peaks in the visible range, and because of the nearly symmetric broadening of the pump spectra, we may ascribe the observed SC mechanism to self-phase modulation. This must occur radially, in order to explain the rainbow spatial distribution of colors. Unfortunately, our numerical model diverges at peak power values well below those necessary to generate a SC covering the whole visible spectral range. Nevertheless, as an alternative approach to explain the origin of colored spiral emission, we simulated the separate propagation of a low peak-power pulse, with different carrier wavelengths across the visible spectral range. As a matter of fact, a colour-separated spiral can be reproduced by the incoherent summation of far-field intensities, independently generated by different pulses with separate wavelengths, as depicted in Fig. 5a. For all spectral components, the fibre length is kept fixed to 1.5 cm, the position offset is 60 $\upmu$m, the beam $1/e^2$ diameter is 11.9 $\upmu$m, $\vartheta =1.5^\circ$, $\varphi =45^\circ$, the input peak power and pulsewidth are 1 kW and 180 fs, respectively. Furthermore, chromatic dispersion is induced by the following wavelength dependence of the cladding refractive index: $n(350\;{\text {nm}}) = 1.4787$, $n(400\;{\text {nm}}) = 1.4701$, $n(500\;{\text {nm}}) = 1.4623$, $n(550\;{\text {nm}}) = 1.4599$ and $n(650\;{\text {nm}}) = 1.4565$. We would like to underline that spirals, obtained numerically for different source wavelengths, all have the same starting point along the spiral arm, as it can be seen in Fig. 2 of the “Supplementary materials”. In each of the plots of Fig. 5a, we only display normalized intensities above the level corresponding to two thirds of the peak value. This permits to highlight, for each wavelength, the respective region of higher intensity along the spiral arm.

### Color separation

**Figure 6:**
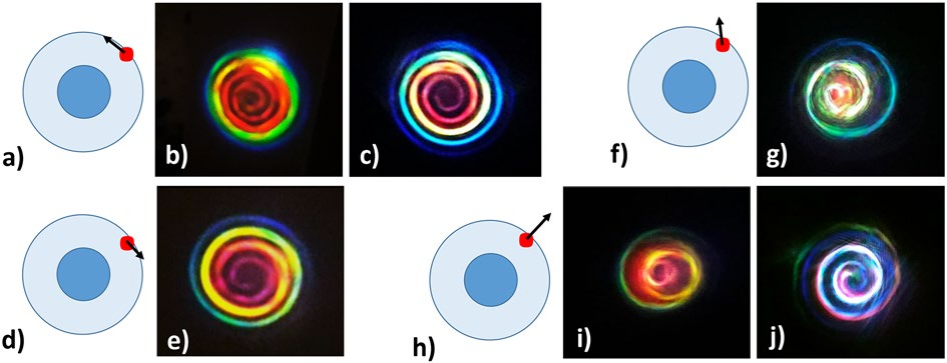
(**a**) Sketch of the input configuration for rainbow spiral emissions obtained in either SI (**b**) or GRIN (**c**) fibres. Black arrows represent the transverse plane components of the input wave vector. (**d**) Symmetric situation with respect to (**a**), leading to flipping the topological charge which results in a change of chirality, as seen in (**e**). (**f**, **g**) Superposition of two spirals with opposite chiralities and different intensities (**g**) (heart-like spiral emission), when the transverse wave vector has a nonzero radial component (**f**). (**h**, **i**) Same as in (**f**, **g**), when the transverse wave vector points along the radial direction. (**j**) Bistable case, resulting in two spirals beam with same chiralities. For all pictures shown, the input power and wavelength were 48 MW and 1030 nm, respectively, and the fibres were 2 cm long. All the images but (**b**) refer to GRIN fibres.

Finally, we could observe that colour spatial separation within the spiral only depends on the input coupling condition, i.e., the position of the incident laser beam on the fibre, while keeping the same spectral composition. In Fig. 5d we show that speckled features appear when the input beam is only partially focused inside the core. By gradually moving deeper into the cladding, spiral emission becomes clearer, and it is characterized by a disordered mix of colours. Finally, when the beam crosses the cladding–air interface, we obtain the rainbow spiral emission, as shown in Fig. 2b-right. In our experiments, multiple arm spirals were also observed. An overview of these hybrid order spiral beams is presented in Fig. 6.

During the experiments, we observed that also the shape of the spiral emission strictly depends on the specific input coupling conditions. A perfect spiral shape configuration is obtained when the input beam wave vector radial component is vanishing. In Fig. 6a we report the particular case of a beam focused on the cladding–air interface: here the black arrow indicates the in-plane component of the wave vector. This configuration gives rise to a rainbow spiral emission in both SI and GRIN fibres, as illustrated in Fig. 6b,c, respectively. If one reverses the direction of the arrow (i.e., by rotating the azimuthal angle), the chirality of the spiral flips its sign (see Fig. 6d,e).

However, one can have intermediate configurations, where the radial component of the wave vector is nonzero. We show in Fig. 6f,g an example of input coupling condition, for which the beam appears as a superposition of spirals with opposite chiralities. One can remark the formation of a heart shape at the centre of the far-field image. In the extreme case, where the transverse component of the wave vector is directed along the radius (Fig. 6h), the two counter-rotating spirals have exactly the same intensity, producing the far-field which is shown in (Fig. 6i). This configuration is bistable, and in the presence of small fluctuations, one may observe the generation of highly complex far-field intensity patterns. Finally, we report in Fig. 6j the case of a two-arms-spiral emission.

## Discussion

The observed Archimedean spiral emission can be explained by the help of the analogy with the generation of angular momentum light beams from single filaments in air^25,26^. This occurs when an intense laser pulse increases the refractive index of air, owing to the Kerr effect. As a consequence, the pulse collapses, and a plasma channel is generated along the optical axis. When beam self-focusing and plasma defocusing compensate for each other, the pulse transforms into a filament, surrounded by an energy reservoir. This is reminiscent of light propagating in optical fibres: the plasma channel acts as the core, and the energy reservoir as the cladding^26^. Upon propagation in the filament, a portion of radiation is lost, being emitted at specific angles, a process which is well known as conical emission^34–40^. Recently, Walter at al. demonstrated angular momentum beam generation by means of deformable mirrors, which permitted to transform a conical emission into spiral emission^25,26^. The latter was ascribed to the difference of group velocity between the plasma channel and the energy reservoir, which allows for a continuous interaction between their modes. The background energy flows helically around the plasma channel (just as seen in Fig. 2c), thus slightly deviating the filament, and impressing a helical path to the beam. In fibres, nonlinearity is not necessary to obtain an optical angular momentum, because the cladding (reservoir) and the core (filament) are defined by the linear index profile. Moreover, no external spatial modulation is needed, since the cylindrical fibre geometry spontaneously creates a TOAM, when symmetry-breaking is seeded by a small tilt of the angle of transverse incidence.

The analogy with radiation emission by single filaments in air allows for an alternative explanation of the mechanism of colour separation in rainbow SC spiral emission from MMFs. In conical emission accompanied by SC generation, shorter wavelengths are emitted with wider angles. The wavelength spread produced by source broadening depends on the Kerr effect-induced electron density gradient: the larger the gradient, the more shifted the generated wavelengths. When SC is generated, its wave vector has a radial component whose modulus linearly grows with optical frequency. This results in a narrower (larger) angle of emission for long (short) wavelengths. In particular, since the radial electron density gradient varies continuously from zero to a maximum value, rainbow rings are generated. This phenomenon has been attributed to self-phase modulation in the radial direction^41–43^.

When applied to our case, the mathematical model to describe the frequency-angular intensity distribution of spectral components $S_{an}(\vartheta ,\lambda ,z)$ is^43^

2 $$\begin{aligned} S_{an}(\vartheta ,\lambda ,z)=S_{0}(\vartheta ,\lambda ,z)l(z)^2 {\text {sinc}}{\frac{\Delta \vartheta }{2}}, \end{aligned}$$

where $\Delta \vartheta$ is the phase excursion of radiation from a broadband point source with distribution $S_0$ and propagating with group velocity $v_g$ over distance $l(z)$ which reads as^43^

3 $$\begin{aligned} \Delta \vartheta =\frac{2\pi l(z)}{\lambda _0}\left[ \left( 1-\frac{\lambda _0}{\lambda }\right) \frac{c_0}{v_g}-\left( 1-\frac{\lambda _0n(\lambda )}{\lambda n_0}\cos (\vartheta )\right) n_0\right] \end{aligned}$$

here $n_0$ is the refractive index, $n(\lambda )$ is material dispersion in silica, and $c_0$ the speed of light in vacuum. Since we spontaneously generate a TOAM beam, the helical path of the photon now leads to rainbow-like spiral emission, instead of a conical emission. The filamentation analogy also helps to explain the colour separation shown in Fig. 5d. Whenever SC generation takes place inside the cladding volume, the pump propagation direction is locally a symmetry axis, and two wavevectors are associated with the same wavelength, which is equivalent to conical emission. This configuration is sketched on the top of Fig. 5e by an intuitive ray-optics picture. The initial spread $2 \Delta \theta$ gets wider after each reflection, leading to an  interference which is responsible for colour mixing in the far-field. One can estimate that very small emission angles ($\Delta \theta < 10^{-5} rad$) are needed in order to incur into interference. Things drastically change when the input beam is focused right at the cladding–air interface (bottom of Fig. 5e). This is because the cladding edge breaks the symmetry along the beam propagation, and a one-to-one correspondence between wavelength and wavevector is imposed by the electromagnetic field continuity condition at the boundary. The resulting lonely ray does not suffer from any interference, and a rainbow colour distribution is observed, as it occurs in the case of conical emission in air.

## Conclusion

We observed the generation of multicolour spiral beam by using standard commercial silica optical fibres. The main trick consists of choosing appropriate fibre coupling conditions, in order to seed a TOAM that, combined with the self-focusing process, permits to generate a large spectral broadening. Our method of spiral beam generation has several advantages. First of all, employing optical fibres allows for easier integration with existing devices and, while conventional LOAM beam generation methods rely on spatial light modulators, here we exploit the conversion of the TOAM, which is intrinsically provided by the cylindrical geometry of the fibre. Moreover, the method is quite robust: it does not depend on the source state polarization, power, and wavelength. The spiral emission formation in fibres is analogous to light filamentation in air. Accordingly, in analogy with conical emission, we could explain the observed rainbow spiral emission in MMFs. Our results provide a substantial advance in structured light generation, and pave the way to the practical application of TOAM beams in data storage, super-resolution, and nanoscale microscopy technologies. Moreover, thanks to the low sensitivity to environmental perturbations with respect to in-air LOAM beam generation, our method can be applied to implement immersible optical tweezers for applications in biology as well.

## Methods

### Experimental setup

Our pulsed light source was a Yb-based laser (Light Conversion PHAROS-SP-HP), generating pulses of 180 fs with 1–100 kHz repetition rate. The laser beam was focused on the input facet of the fibre, and incident with controllable tilt angles $\vartheta$ and $\varphi$ with respect to the fibre axis *z*, and with a beam diameter of about 10 ${\upmu }$m at $1/e^2$ of peak intensity. Both the parabolic GRIN fibre and the step-index fibre had a core radius $r_{\text {c}}=25$ ${\upmu }$m, cladding radius 62.5 ${\upmu }$m, and a cladding index $n_{\text {clad}}=1.45$ at $\lambda = 1030$ nm. The relative core–cladding index difference was $\Delta =0.0103$ or 0.0120 for the GRIN and the step-index fibre, respectively. The index grading is realized by Germanium doping. The top view images in Fig. 2c–e were taken with a Dinolight2.0 digital microscope for 14 mW average input power from the femtosecond laser at 1030 nm, at the repetition rate of 2 kHz. Spectra were collected by a miniature fibre optics spectrometer (Ocean Optics USB2000+), with 200–1100 nm spectral range and an optical spectrum analyzer (OSA) (Yokogawa AQ6370D), while far and near-field images were taken by a Reflex digital camera (Nikon D850) and a Gentec Beamage-4M-IR CCD camera, respectively. Input and output average powers were measured by a thermopile power meter (GENTEC XLP12-3S-VP-INT-D0). Our CW light source was a 4 mW HeNe-based laser (Thorlabs HNLS008L-EC).

### Numerical model

The numerical model developed to describe the process of spiral emission generation in MMFs is the $(3D+1)$ GNLSE (or Gross–Pitaevskii equation^44^) in its vectorial form, involving a single field for each polarization, including all frequencies and modes^33^:

4 $$\begin{aligned} \frac{\partial A_p(x,y,z,t)}{\partial z}&=\frac{i}{2k} \left( \frac{\partial ^2A_p}{\partial x^2}+\frac{\partial ^2 A_p}{\partial y^2}\right) \nonumber \\&\quad -i\frac{\beta _2}{2}\frac{\partial ^2A_p}{\partial t^2}+ \frac{\beta _3}{6}\frac{\partial ^3A_p}{\partial t^3}+ i\frac{\beta _4}{24}\frac{\partial ^4A_p}{\partial t^4} -\frac{\alpha }{2}A_p+\nonumber \\&\quad +i\frac{k}{2}\left[ \frac{n^2(x,y)}{n^2_{0}}-1\right] A_p+ i\gamma \left( 1+iK_2+\frac{i}{\omega _0}\frac{\partial }{\partial t}\right) \left[ (1-f_R)A_p\left( |A_p|^2+\frac{2}{3}|A_q|^2+\frac{1}{3}A_p^2e^{-2i\omega _0t}\right) \right. \nonumber \\&\left. \quad + f_RA_p\int _{-\infty }^t d\tau h_R(\tau )\left( |A_p(t-\tau )|^2+\frac{2}{3}|A_q(t-\tau )|^2\right) \right] \end{aligned}$$

with $k=n_0\frac{2\pi }{\lambda }$, $\gamma =n_2\frac{2\pi }{\lambda }$, $K_2=\frac{\alpha _2}{2\gamma }$, and $f_R=0.18$. In Eq. (4), the two polarizations $p,q=x,y$ are nonlinearly coupled. Terms in the right-hand side of Eq. (4) account for: transverse diffraction, second, third, and fourth-order dispersion, linear loss, the waveguiding term with refractive index profile *n*(*x*, *y*) and core index $n_\text {0}$, Kerr and Raman nonlinearities (with nonlinear coefficient $\gamma$ and fraction $f_R$), respectively. In Eq. (4) we neglect the contribution of polarization mode dispersion: we numerically verified that its effects are negligible for the short fibre lengths (few centimeters) involved in our experiments. Nonlinearities include self-steepening, third-harmonic generation (THG), and two-photon absorption (TPA) (with coefficient $\alpha _2$).

In simulations, we used the following GRIN fibre parameters: core radius, cladding radius, and relative index difference are already provided in the experimental setup subsection; dispersion parameters are $\beta _2=18.9\,{\text {ps}}^2/{\text {km}}$ at 1030 nm, $\beta _3=0.041\,{\text {ps}}^3/{\text {km}}$, $\beta _4=-5.3\times 10^{-5}\, {\text {ps}}^4/{\text {km}}$; nonlinear parameters are $n_2=2.7\times 10^{-20}\, {\text {m}}^2/{\text {W}}$, $\alpha _2=1\times 10^{-16}\, {\text {m}}/{\text {W}}$; $h_R(\tau )$ with the typical response times of 12.2 and 32 fs, respectively^45,46^. We included the wavelength dependence of the linear loss coefficient $\alpha$, as reported for standard SM glass fibres^47^. The input beam was modeled as a Gaussian beam with $w_0=5$ $\upmu$m waist (10 $\upmu$m diameter); we used a Gaussian temporal shape with full-width-at-half-maximum (FWHM) pulse-width $T_{\text {FWHM}}=180$ fs. The input beam was coupled at different points of the cladding or the core, and tilted by small angles (up to $15^{\circ }$).

## Supplementary information

Supplementary Video 1.

Supplementary Video 2.

Supplementary Video 3.

Supplementary Video 4.

Supplementary Information.
