# Supplementary material for: Rainbow Archimedean spiral emission from optical fibres
Source: Sci Rep. 2021 Jun 22;11:13030. doi: 10.1038/s41598-021-92313-w (PMC8219727; doi:10.1038/s41598-021-92313-w)
Supplement: Supplementary file 5 — Supplementary Information. [file 41598_2021_92313_MOESM5_ESM.pdf]

Rainbow Archimedean spiral emission from optical fibres

F. Mangini[1]∗, M. Ferraro[2]∗, M. Zitelli[2]∗, V. Kalashnikov[2], A. Niang[1], T. Mansuryan[3], F. Frezza[2], A. Tonello[3], V. Couderc[3], A.B. Aceves[4], S. Wabnitz[2]

[1]*Department of Information Engineering (DII), University of Brescia, Via Branze 38, 25123 Brescia, Italy.*

[2]*Department of Information Engineering, Electronics and Telecommunications (DIET), Sapienza University of Rome, Via Eudossiana 18, 00184 Rome, Italy.*

[3]*Université de Limoges, XLIM, UMR CNRS 7252, 123 Avenue A. Thomas, 87060 Limoges, France*

[4]*Department of Mathematics, Southern Methodist University*

∗ *These authors have contributed equally*

Supplementary

Spiral emission as a function of input pulse duration, peak power, and wavelength. The objective of this section is to show that spiral emission by injecting a laser beam in an optical fibre is not affected by a change of different parameters of the source beam. We demonstrate that, by showing images of the far-field at the fibre output, when we vary either the peak power, the time duration of the incident laser pulse, or the source wavelength. Fig. 1a and b show far-field (first two rows) and near-field images (third row) at the output of 2 cm long GRIN or SI fibres. The near-field is only reported for the GRIN fibre case. Here the wavelength and the pulse duration are kept at 1030 nm and 180 fs, respectively, while the input peak power is varied between 0.1 MW and 6 MW. These values remain below the SC generation threshold, so that spiral emission is only observed in the near-IR (i.e., at the pump wavelength). By comparing the different images, we can see that that only intensity variations occur (owing to the increase of input power), while the spiral shape remains unchanged. Fig. 1b shows the evolution of the far-field intensity from the output of a 2 cm length of GRIN MMF at 1030 nm, with 0.1 mW of input average power, when the pulse duration is varied from 180 ps to 8 ps. As we can see, spiral generation is independent of the time duration of the input pulse as well, which confirms the linear nature of the phenomenon.

We confirmed that spiral beams can always be obtained, also when the pump wavelength is varied from 1030 nm. In Fig. 2a and b we show the far-field at the output of GRIN and SI fibres, respectively, when the input wavelength ranges from 650 nm to 940 nm, while keeping the same incidence angle and position of the input beam. In Fig. 2c and d we report the corresponding near-field distributions and spectra for the case of a GRIN fibre only. Again, we can remark that the formation of far-field spiral shapes is always observed, for any value of the source wavelength. In Fig.2e we report the spectra obtained from GRIN MMFs, when varying the input peak power up to 49 MW. In Fig.2f, we experimentally confirm that under the same laser/fiber coupling conditions, the length of the spiral arm increases as the fibre length grows larger. The maximum length of the fibre from which a spiral emission is obtained is about 5 cm. After this length, the phase distribution of the beam inside the fibre is no longer able to guarantee spiral emission.

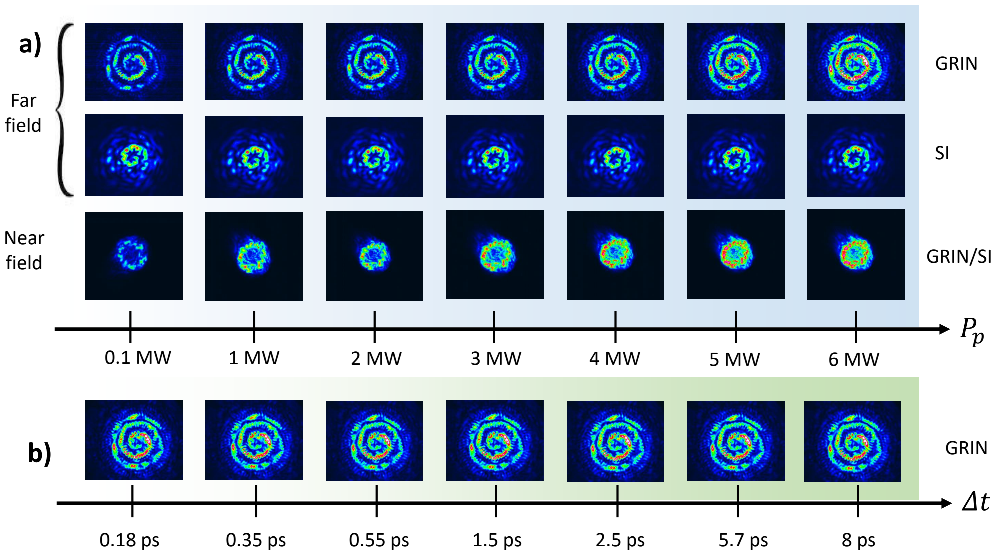

Figure 1: (a) Near and far-field images at the output of a 2 cm GRIN or SI fibre. The input peak power varies from 0.1 MW to 6 MW, with a repetition rate 50 kHz. The source wavelength is 1030 nm, while the fibres are 1.5 cm long. The coupling angles are $\vartheta = 2°$ and $\varphi = 45°$. (b) Far-field images from GRIN fibre, under the same conditions as (a). Here the input average power is maintained at 0.1 mW, while the pulse duration varies between 0.18 to 8 ps

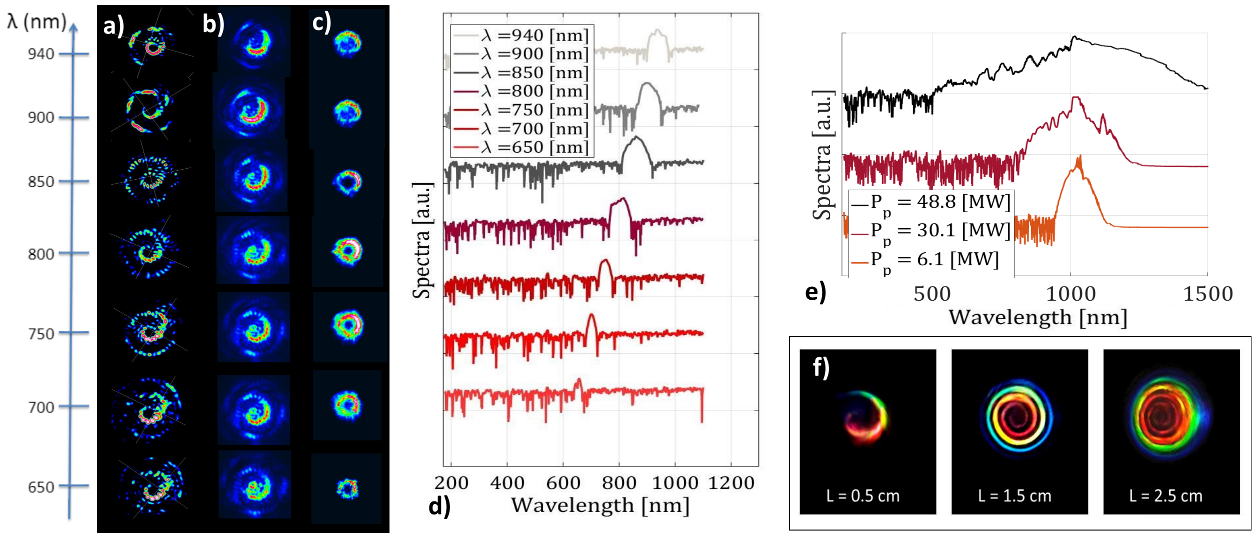

Figure 2: (a) Far-field images from a 2 cm long GRIN when the wavelength of the laser source varies between 650 and 940 nm. The input power is kept at 0.1 MW, with a 50 kHz repetition rate; the input beam coupling angles are $\vartheta = 2°$ and $\varphi = 45°$. (b) Same as in (a), for a SI fibre. (c-d) Near-field output intensity and spectra corresponding to (a). (e) fibre output spectra at different input peak powers (up to 48 MW) at 1030 nm in GRIN fibres keeping the same coupling conditions. (f) Rainbow spiral emission from different lengths of GRIN fibre: 0.5 cm, 1.5 cm, and 2.5 cm, respectively.

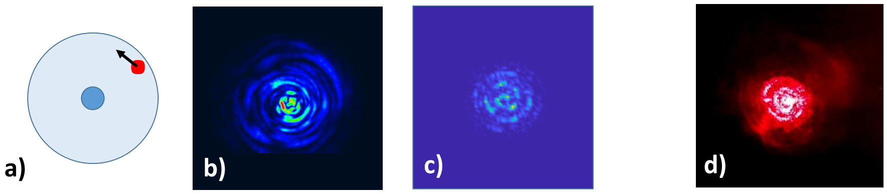

Figure 3: (a) Sketch of the input coupling configuration for spiral emissions in an SMF. Black arrows represent the in-plane component of the input wave vector. (b) Experimental and (c) numerical far-field images from a 2 cm long SMF at 1030 nm. The input peak power is 0.1 MW with a repetition rate of 50 kHz, and the coupling angles are $\vartheta = 2°$ and $\varphi = 45°$. (d) Spiral emission from a commercial laser pointer.

Spiral emission from singlemode fibre and from laser pointer The objective of this section is to demonstrate, both experimentally and numerically, that spiral emission from a singlemode fiber (SMF) only occurs when injecting the beam inside the cladding. Fig. 3a schematically illustrates the input coupling condition. The laser beam is offset by about 45 μm with respect to the fibre axis, with $\vartheta = 1.5°$ and $\varphi = 45°$. Figs. 3b,c show the far-field image at the output of a 2 cm long SMF, as obtained either experimentally or numerically, under the same conditions. The wavelength and the pulse duration were kept at 1030 nm and 180 fs, respectively, while the input peak power was 0.1 MW, and the repetition rate was 50 kHz. As can be seen from these images, even in the case of a singlemode core it is possible to generate spiral emission out of the fibre, by suitably coupling the incoming laser beam with the fibre cladding. Additionally, in Fig. 3d we report the far-field image from GRIN MMFs, using a commercial laser pointer.
